# 4D-Micro: a tiered, testable framework for hemodynamic-tissue perfusion mismatch in cardiac surgery

**DOI:** 10.3389/fcvm.2026.1964275

**Published:** 2026-09-30

**Authors:** Budanbaila La, Yanyan Bai, Yiri Du, Yi Qiu, Ling Wang

**Affiliations:** 1 Department of Anesthesiology, The Affiliated Hospital of Inner Mongolia Medical University, Hohhot, Inner Mongolia, China; 2 Department of Anesthesiology, Hohhot Maternal and Child Health Hospital, Hohhot, Inner Mongolia, China; 3 Department of Anesthesiology, The Second Affiliated Hospital of Inner Mongolia Medical University, Hohhot, Inner Mongolia, China

**Keywords:** cardiac surgery, cardiopulmonary bypass, hemodynamic coherence, implementation, microcirculation, state estimation, validation, venous congestion

## Abstract

Organ dysfunction can develop after cardiac surgery even when mean arterial pressure, cardiac output, hemoglobin, temperature, and pump flow meet conventional targets. These variables remain the safety foundation, but they do not directly establish exchange-capable capillary flow or cellular oxygen use. We propose 4D-Micro, an unvalidated conceptual framework organized around four necessary functions: barrier and conduit integrity, perfusion driving, hemorheology, and cellular oxygen use. The framework was formalized from a recurrent clinical reasoning problem through targeted evidence mapping and explicit construction rules; it was not derived from a new patient dataset or statistical latent-variable analysis. 4D-Micro separates where recovery is discordant from the provisional mechanism that might explain it, then uses a prespecified proximal response and stopping boundary to update that explanation. It is not a substitute for established cardiac surgical monitoring or a claim that all four domains can be measured directly at the bedside. A tiered implementation distinguishes a core mode based on routine variables, an enhanced mode using regional and congestion signals, and a research mode using direct microvascular or biomarker measurements. Insufficient or conflicting data remain indeterminate rather than being forced into a phenotype. We specify a staged validation pathway: content and feasibility testing, prospective assessment of classification reliability and construct validity, and only then a pragmatic comparison with protocolized usual hemodynamic care. The framework should be narrowed or rejected if it cannot be applied reliably, its predicted responses are not observed, or it fails to improve decision processes without added harm.

## 1 Introduction

Hemodynamic management during cardiac surgery must begin with MAP, CO/CI, CPB pump flow, hemoglobin, arterial oxygen saturation, temperature, acid-base status, and echocardiographic assessment when indicated. Serious abnormalities in these variables threaten perfusion and require prompt correction. The narrower question addressed here arises after that safety work has begun: do systemic recovery and tissue-level recovery remain aligned? Acceptable pressure and bulk flow cannot exclude heterogeneous capillary red-cell transit, endothelial edema, a reduced arterial-to-venous gradient, or impaired cellular oxygen use (1–3).

Loss of agreement between macrocirculation and tissue perfusion is often described as loss of hemodynamic coherence (1, 2). Cardiac surgery makes this disagreement difficult to interpret because physiology can change within minutes. CPB combines hemodilution, artificial surfaces, altered shear, temperature change, inflammation, coagulation activation, ischemia-reperfusion, and vasoplegia. OPCAB avoids some bypass exposures but introduces cardiac displacement, right-ventricular compression, transient coronary occlusion, reduced venous return, and event-linked regional ischemia (4–9). The operative label therefore does not specify the active limiting mechanism.

Recent reviews already describe perioperative microcirculatory physiology, CPB-related injury, monitoring, and candidate protective strategies (3, 4, 10, 11). 4D-Micro does not repeat those reviews as a new treatment protocol. It proposes a testable structure for five unresolved decisions: how to recognize cross-layer discordance, separate its location from its suspected cause, state uncertainty when data are incomplete, use only clinically indicated interventions as bounded physiologic tests, and measure whether this structure adds value beyond protocolized usual care.

## 2 Conceptual origin, construction rules, and evidence boundaries

The framework originated from a recurrent clinical reasoning problem: systemic variables may improve while regional, microvascular, metabolic, or organ signals do not. Clinical experience motivated that question but was not treated as validation data. We formalized the proposal through targeted, iterative searches of PubMed/MEDLINE and the Cochrane Library, updated through August 5, 2026. Search blocks combined cardiac surgery, CPB, or OPCAB with microcirculation, endothelial barrier and glycocalyx, oxygen delivery, NIRS, HVM, carbon-dioxide gaps, lactate, vasoplegia, venous congestion, ultrafiltration, and patient blood management. Current guidelines, systematic reviews, pivotal trials, and reference lists were used to test whether each proposed construct had physiologic support. Full search concepts and selection priorities are provided in Supplementary Table S1.

Construction followed four prespecified rules. First, each domain had to represent a necessary function in tissue oxygen exchange that could fail despite correction of another domain. Second, a coherence state had to describe where recovery diverged, whereas a phenotype had to describe a potentially reversible explanation; the two could not be interchangeable. Third, a phenotype was retained only if it generated a directional, time-linked proximal response that could be sought after a safe and clinically indicated intervention. Fourth, the framework had to permit mixed and indeterminate classifications. These rules and rejected alternatives are listed in Supplementary Table S2.

We separated three evidence levels throughout the model: mechanistic plausibility, evidence that a proximal physiologic response can occur, and evidence for patient-important benefit. A plausible mechanism does not establish clinical utility, and improvement in a monitor does not prove benefit. Numerical values are therefore triggers for reassessment, not universal treatment thresholds. Recommendations supported by established cardiac-surgery guidance remain distinct from the investigational classification and response-prediction components of 4D-Micro.

This is a theory-construction exercise, not a systematic review, diagnostic-accuracy study, or treatment trial. There was no registered protocol, exhaustive dual screening, formal risk-of-bias assessment, meta-analysis, original patient dataset, or statistical derivation of latent classes. The four domains, four coherence states, and six phenotypes are author-proposed constructs. They should not be presented or used as validated bedside categories before prospective testing.

## 3 Four functional domains and the proposed contribution

Effective tissue exchange requires four linked functions (Figure 1). The barrier and conduit domain (B) concerns recruited exchange surface, permeability, and diffusion distance. Perfusion driving (D) concerns forward flow, the arterial-to-venous pressure gradient, shear, and regional distribution. Hemorheology (R) concerns delivery of oxygen-bearing red cells without marked stasis, loss of deformability, or obstruction. Cellular oxygen use (U) concerns diffusion, extraction, mitochondrial oxidation, and metabolic recovery. We use x(t) = [B(t), D(t), R(t), U(t)]ᵀ only as conceptual notation; it is not a validated score or bedside equation.

**Figure 1:**
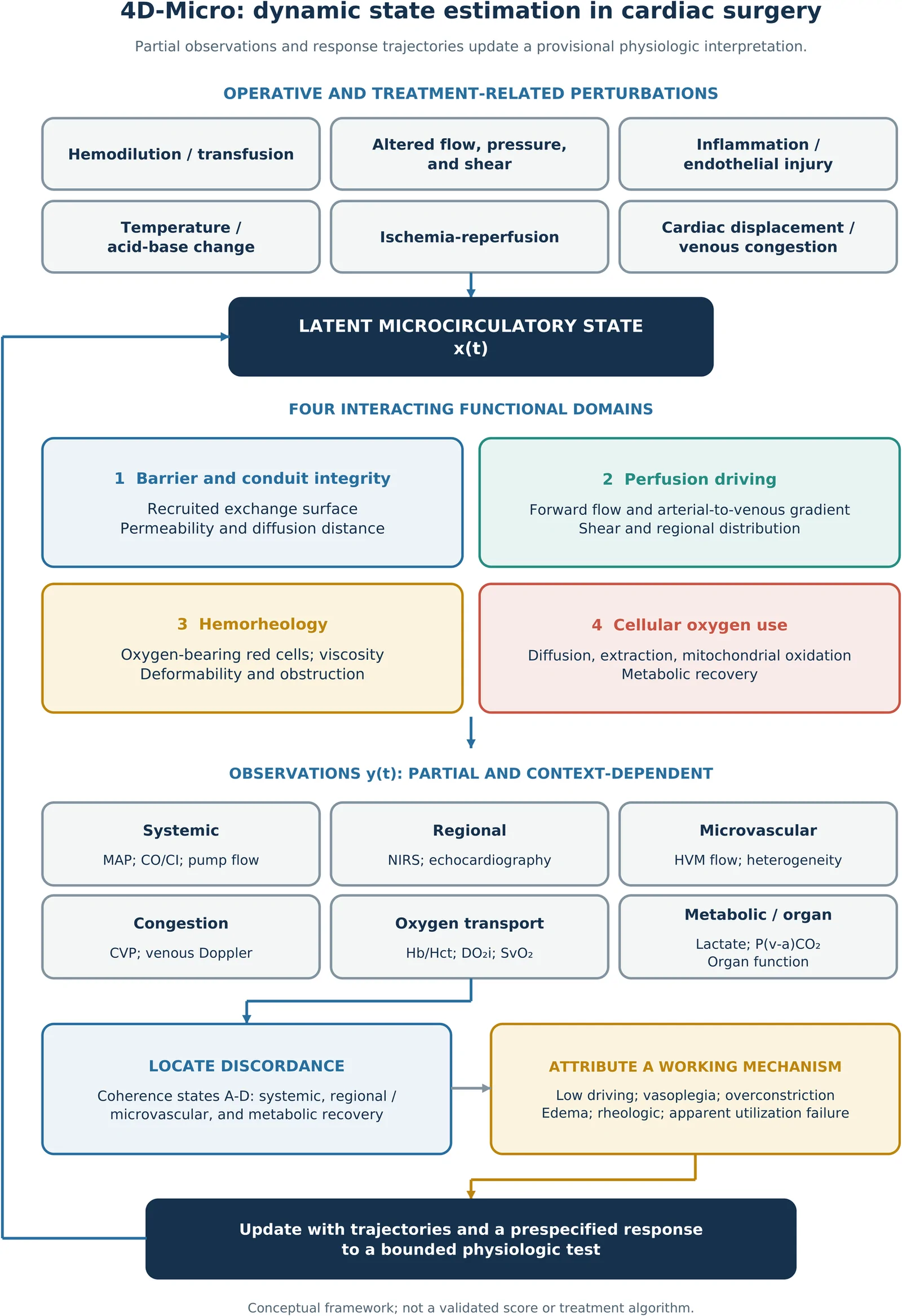
Dynamic state estimation with 4D-Micro during cardiac surgery. Operative events and treatments alter a latent physiologic state described by four interacting domains. Bedside variables are partial observations whose meaning depends on context. The coherence state locates discordant recovery, while the provisional phenotype suggests a mechanism. Signal trajectories and the response to a bounded physiologic test update confidence. HVM and biomarker observations are optional research-level measures, not prerequisites for the core workflow.

The domains interact rather than forming independent compartments. Barrier injury can produce edema and lengthen the diffusion path; venous congestion narrows the effective perfusion gradient; slow flow favors leukocyte adhesion, red-cell aggregation, and microthrombosis; reperfusion-related oxidative stress and disturbed calcium handling may injure mitochondria and endothelium (12–16). A response in one signal therefore cannot by itself identify the limiting domain.

Bedside variables are partial observations of these functions. MAP, CO/CI, pump flow, hemoglobin/hematocrit, indexed oxygen delivery (DO_2_i), SvO_2_/ScvO_2_, NIRS, HVM, echocardiography, venous pressure or Doppler, P(v-a)CO_2_, lactate, and organ function differ in anatomic scale, delay, and susceptibility to artifact. The framework therefore uses within-patient trajectories, cross-layer agreement, operative context, and response to a focused test rather than assigning a domain from one threshold.

Time is part of the hypothesis. A short disturbance that resolves after repositioning differs from a persistent or recurrent disturbance. Nadir MAP, nadir DO_2_i, or peak lactate omits duration, recurrence, and completeness of recovery. Candidate burden measures include time below an individualized DO_2_i range, area of NIRS deviation from baseline, recurrent impaired HVM flow where measured, duration of congestion, oxygen-extraction trajectories, and metabolic recovery slopes (17). None is yet a validated 4D-Micro treatment target.

The proposed contribution is therefore not discovery of four new mechanisms, nor a claim that experienced clinicians do not already treat and reassess. It is the combination of five explicit rules: separate the location of discordant recovery from its suspected cause; require a trajectory across physiologic layers; preserve an indeterminate category; prespecify the response and stopping boundary before escalation; and define observations that would refute the working explanation.

## 4 Tiered implementation and minimum classification rules

Implementation is tiered so that the framework does not depend on universal access to HVM, advanced NIRS, biomarker panels, or a single proprietary platform (Table 1). Tier 0 is the conventional safety platform: operative phase and event time, MAP, pump flow or CO/CI when available, hemoglobin and arterial oxygenation, temperature, acid-base status, venous pressure, serial metabolic trends, urine output, and echocardiography when clinically indicated. These data may raise suspicion of mismatch, but they do not directly diagnose microcirculatory failure. Tier 1 adds available regional and congestion information, such as NIRS, P(v-a)CO_2_, or structured venous Doppler. Tier 2 includes HVM, glycocalyx biomarkers, and other direct or experimental measurements and is intended primarily for validation studies (18–23).

**Table 1:** Four domains and tiered observation strategy.

**Domain**	**Core function**	**Tier 0: conventional safety data**	**Tier 1: enhanced observations**	**Tier 2: research observations**
Barrier and conduit	Exchange surface, permeability, diffusion distance	Fluid balance; oxygenation; lung mechanics; examination; echocardiographic context	Structured venous Doppler; lung-water estimate where available; repeated congestion assessment	HVM; glycocalyx markers; endothelial or permeability panels
Perfusion driving	Forward flow, effective arterial-to-venous gradient, shear, regional distribution	MAP; pump flow or CO/CI; CVP; arterial gas; TEE when indicated	NIRS; detailed venous Doppler; continuous flow or pressure trending	HVM flow and heterogeneity; synchronized multi-organ measurements
Hemorheology	Delivery of oxygen-bearing red cells without marked stasis or obstruction	Hb/Hct; DO_2_i; temperature; acid-base status; bleeding and coagulation assessment	Viscoelastic testing; serial oxygen-extraction data	Red-cell deformability, viscosity, or microthrombotic assays
Cellular oxygen use	Diffusion, extraction, oxidation, and metabolic recovery	Lactate trajectory; SvO_2_/ScvO_2_ when available; urine output and organ function	P(v-a)CO_2_; region-specific recovery trends	Microdialysis; metabolic or mitochondrial phenotyping

A minimum viable bedside record contains the operative event, a baseline or immediately preceding value, at least one systemic flow/pressure measure, oxygen-carrying capacity, one non-systemic or metabolic observation when available, the intervention, the predicted response, the reassessment window, and the stop boundary. Classification should use at least two time points and should not be made from a single unverified value. If cross-layer information is absent, internally conflicting, or dominated by artifact, the case is recorded as indeterminate. Missing measurements lower confidence; they are not assumed to be normal.

The four domains are not a checklist that must all be measured simultaneously. The clinician first identifies which available layer is failing to recover, then states one dominant or mixed mechanism with a confidence level. A research case-report form and minimum data dictionary are provided in Supplementary Tables S3 and S4. This limited workflow is the unit to be tested; clinical adoption should not be inferred from conceptual plausibility alone.

## 5 CPB and OPCAB as trajectories, not fixed states

CPB and OPCAB are treated as different trajectories through a shared perturbation space, not as fixed phenotypes. CPB usually produces a broad exposure to hemodilution, artificial surfaces, altered shear, hypothermia, inflammation, coagulation activation, and ischemia-reperfusion. Unclamping and rewarming may add vasoplegia, endothelial swelling, oxidative stress, and heterogeneous flow. Microvascular and metabolic recovery can therefore lag behind normalization of MAP and CO (2–4, 12–15).

During OPCAB, elevation, rotation, and stabilization of the heart can reduce venous return, compress the right ventricle, lower CO, and create event-linked regional ischemia. Sublingual and cerebral signals may worsen with displacement and improve after repositioning (5), while cerebral and peripheral oxygenation may relate differently to MAP and CI (7). The informative unit is the trajectory before, during, and after each displacement or occlusion, including whether recovery is complete.

This framework does not imply superiority of either technique. Long-term randomized evidence has not established a universal advantage of OPCAB over CPB, and outcomes depend on expertise, conversion, and completeness of revascularization (8, 9). Mechanical correction and surgical safety take precedence over framework classification. Persistent low flow, regional ischemia, right-heart compromise, or threatened graft quality may appropriately lead to conversion; a brief fully reversible event does not carry the same implication.

## 6 Coherence states and provisional mismatch phenotypes

A coherence state locates discordant recovery (Table 2). State A is concordant recovery across the layers that are actually observed. State B is macro-micro discordance: pressure and forward flow recover while a direct microvascular measure or corroborated non-systemic signals remain impaired. State C is focal regional discordance: global variables and other observed regions are stable, but one event-linked organ or regional signal worsens. State D is delayed metabolic or organ recovery after delivery and reversible regional causes have been reassessed. State D is not synonymous with mitochondrial failure. Adrenergic lactate, hepatic clearance, congestion, occult ischemia, and inadequate effective delivery remain alternatives.

**Table 2:** Operational definitions of coherence states and the indeterminate category.

**Classification**	**Operational description**	**Minimum corroboration**	**Confidence-lowering conditions**
State A: concordant recovery	Systemic and all available non-systemic/metabolic layers improve over the relevant event window	At least two time points and no persistent opposing signal	Few observed layers; delayed markers not yet interpretable
State B: macro-micro discordance	Pressure/flow recover, but a direct microvascular measure or corroborated non-systemic signals remain impaired	Direct HVM abnormality or two compatible non-systemic observations	No direct measure; organ disagreement; major artifact
State C: focal regional discordance	Global variables and other observed regions are stable, but one event-linked organ/regional signal worsens	Reproducible event linkage or second organ-specific signal	Sensor displacement; extracranial contamination; unresolved mechanical cause
State D: metabolic/organ lag	Delivery and reversible regional causes have been reassessed, yet metabolic or organ recovery remains delayed	Serial trajectory over a biologically plausible interval	Adrenergic lactate; hepatic dysfunction; occult ischemia or congestion
Indeterminate (safety category)	Data are insufficient, conflicting, or dominated by artifact	No forced classification	Missing cross-layer data or unresolved urgent cause

A mismatch phenotype proposes cause rather than location (Table 3). The six working labels are low-driving mismatch, vasoplegia, microvascular overconstriction, barrier leak/edema, rheologic or microthrombotic mismatch, and apparent utilization failure. They are not disease diagnoses. More than one may coexist, and no label should be assigned solely because a treatment commonly associated with it was given.

**Table 3:** Provisional mismatch phenotypes and bounded response tests.

Provisional phenotype	Pattern that raises suspicion	Priority exclusions	Bounded test and predicted proximal response
Low driving	Low flow/pump flow, high venous pressure, falling NIRS, widened P(v-a)CO_2_	Bleeding; RV compression/failure; tamponade; circuit or mechanical problem	Correct mechanics, flow, effective preload, or venous back pressure; expect compatible improvement in flow and a non-systemic signal
Vasoplegia	Low resistance with relatively preserved flow and increasing vasopressor need	Hypocalcemia; acidosis; hypothermia; severe myocardial dysfunction	Evidence-based vasopressor treatment; expect pressure recovery without loss of regional perfusion; stop for ischemia, toxicity, or absent response
Microvascular overconstriction	Adequate/high MAP with cold periphery or poor regional/direct microvascular trend	Low flow; hypothermia; pain; mechanical cause	Reduce avoidable catecholamine burden and correct flow/temperature/analgesia; expect non-systemic improvement without instability
Barrier leak/edema	Positive fluid balance, edema/congestion, worsening oxygenation	True intravascular depletion; uncontrolled bleeding	Avoid ineffective fluid; decongest selectively if indicated; expect congestion/oxygenation improvement while flow is preserved
Rheologic/microthrombotic	Low Hb/DO_2_i, heterogeneous flow, bleeding or specific coagulation abnormality	Hypothermia; acidosis; surgical bleeding	Apply patient blood management and treat a defined deficit; a higher Hb without physiologic improvement is insufficient
Apparent utilization failure	Reasonable delivery with persistent metabolic or organ dysfunction	Regional ischemia; congestion; adrenergic lactate; hepatic dysfunction	Avoid repeated ineffective escalation; support organs and reassess causes; deterioration reopens the search

State and phenotype answer different questions. For example, State B may reflect excessive vasoconstriction, congestion, rheologic disturbance, or a mixture. The same phenotype can appear in several states as the operation evolves. Every label is therefore provisional, time-stamped, and revisable. An indeterminate designation is preferable to false precision when signals disagree or key layers are unavailable.

## 7 Response-guided reasoning and clinical safeguards

The response-guided cycle is triggered by new discordance, a worsening trajectory, failed recovery, or an organ warning (Figure 2). The first step is not phenotype assignment. The signal is verified, and bleeding, circuit failure, mechanical obstruction, RV compression, tamponade, major arrhythmia, ischemia, ventilation problems, and other urgent causes are addressed. Only then is a dominant or mixed mechanism stated. Any intervention used as a physiologic test must already be clinically reasonable, limited in dose or duration, and accompanied by a prespecified proximal response, reassessment window, and toxicity/stop boundary. A fluid bolus, vasopressor escalation, or other potentially harmful treatment should never be given solely to complete the classification.

**Figure 2:**
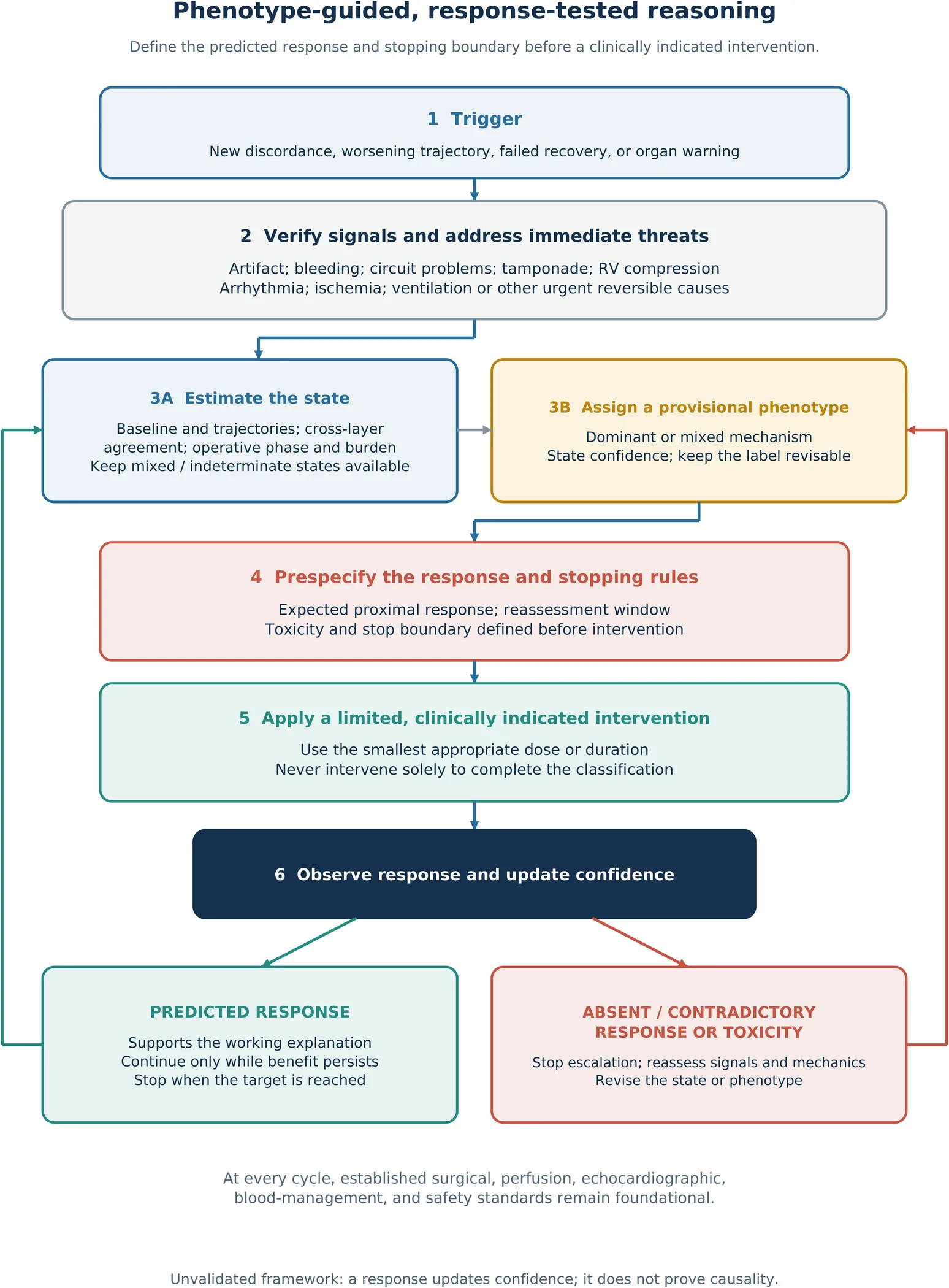
Clinician-directed, response-guided reasoning. A new or worsening discordance is checked for artifact and urgent reversible causes before a working state and provisional mechanism are assigned. Only a clinically reasonable, limited intervention is used to test the suspected constraint. The predicted proximal response, reassessment window, and stopping boundary are defined before escalation. An absent, contradictory, or toxic response prompts cessation and reclassification.

For a low-driving hypothesis, the expected response may be improvement in CO/CI, regional oxygenation, microvascular flow where available, or P(v-a)CO_2_ after correction of flow, effective preload, mechanics, or venous back pressure. A higher MAP with unchanged or worse non-systemic signals weakens the claim that arterial pressure was the main constraint. The observation updates confidence; it does not prove a unique mechanism because most interventions have several physiologic effects.

Treatment remains grounded in existing evidence rather than in the framework. In confirmed low-resistance vasoplegia, norepinephrine is conventional; vasopressin can reduce catecholamine exposure in selected patients but is not indicated for every episode of hypotension (24–26). Methylene blue and hydroxocobalamin remain rescue options for refractory cases with explicit response and toxicity criteria (27, 28). These examples illustrate a response test; they are not new drug recommendations from 4D-Micro.

For barrier leak or congestion, additional fluid may improve exchange when preload is truly inadequate, but can worsen hydrostatic pressure, venous back pressure, and diffusion distance. Albumin has not shown routine superiority to Ringer acetate for major outcomes; priming composition can alter proximal microcirculatory physiology without establishing broad outcome benefit; and ultrafiltration has not provided universal renal or survival protection (29–32). Decongestion is therefore a mechanism-matched option only when congestion is demonstrated and forward flow can be preserved.

Rheologic decisions should remain within contemporary patient blood management, integrating hemoglobin, DO_2_i, temperature, acid-base status, bleeding, coagulation, and transfusion risk rather than pursuing an arbitrary hemoglobin value (33). Persistent metabolic abnormality after supply and regional causes have been reassessed does not justify indefinite escalation. Hyperoxia increased oxidative stress without reducing organ injury in the ROCS trial (34). Goal-directed perfusion, autoregulation-guided pressure, pulsatile perfusion, MiECC, corticosteroids, and monitor-guided strategies retain their own evidence boundaries (35–39); 4D-Micro does not upgrade that evidence.

## 8 Staged validation, comparator, and falsification criteria

Validation should follow the staged logic used for complex clinical interventions rather than proceed directly to an outcome trial (40) (Table 4). Stage 0 should use multidisciplinary co-design, cognitive interviews, and standardized vignettes to test whether definitions are understandable, whether clinicians can identify an indeterminate case, and whether the documentation burden is acceptable. Failure at this stage should trigger simplification rather than device expansion.

**Table 4:** Staged validation and explicit comparators.

Stage	Design and purpose	Prespecified comparator	Decision criterion
0. Content and feasibility	Co-design, cognitive interviews, standardized vignettes; refine definitions and workflow	Independent expert reasoning and alternative labels	Acceptable comprehension and burden; reliable recognition of indeterminate cases
1. Reliability and construct testing	Prospective synchronized cohort; independent blinded classification; nested advanced monitoring	Between-rater agreement and predicted vs non-predicted responses	Adequate classifiability, time burden, agreement, and directional response consistency
2. Incremental observational validity	Multicenter longitudinal cohort; state duration, recurrence, transitions, recovery kinetics	Baseline risk plus time-varying conventional variables	Reproducible, calibrated incremental information; not association alone
3. Comparative clinical utility	Cluster-randomized or stepped-wedge workflow evaluation	Protocolized usual hemodynamic care with identical safety monitoring and rescue access	Less unresolved discordance and ineffective treatment without greater delay, burden, or harm; clinical outcomes only after feasibility and safety

Stage 1 should be a prospective feasibility and reliability cohort with synchronized event markers and repeated observations. Two or more clinicians, blinded to outcomes and to each other's labels, should classify identical episodes. Primary measures are the proportion of episodes classifiable with Tier 0 or Tier 1 data, time needed, interobserver agreement, frequency of mixed/indeterminate labels, and whether prespecified proximal responses occur more often when the assigned mechanism predicts them. Advanced HVM or biomarker measurements can be nested at capable sites rather than required for all centers.

Stage 2 should test construct and incremental prognostic validity in a multicenter cohort. The prespecified comparator is a conventional model containing baseline surgical risk and time-varying MAP, flow/CI or pump flow, DO_2_i, hemoglobin, temperature, vasoactive exposure, lactate, and major operative events. The candidate addition is state duration, recurrence, transition, confidence, and recovery kinetics. The framework is informative only if these variables show reproducible associations and add calibrated information beyond the comparator; association alone would not establish clinical utility.

Stage 3 should compare the structured workflow with protocolized usual hemodynamic care using the same mandatory safety monitoring and access to rescue treatment in both groups. A cluster-randomized or stepped-wedge design may reduce contamination. Initial outcomes should be process and safety measures: time to recognition, time in unresolved discordance, repeated ineffective or contradictory interventions, protocol burden, hypotension exposure, arrhythmia, ischemia, fluid balance, and transfusion. A later adequately powered trial could examine organ and patient-centered outcomes only if reliability and safety are acceptable.

The framework is falsified or requires major revision if clinicians cannot apply it reliably; most episodes remain indeterminate; proposed states do not show distinct trajectories; mechanism-concordant responses are no more frequent than responses under alternative labels; mismatch burden adds no information beyond conventional variables; or framework-guided care increases delay, intervention burden, or harm. The protocol-ready hypotheses and a worked hypothetical example are provided in Supplementary Tables S5 and S6.

## 9 Discussion

4D-Micro is deliberately narrower than a comprehensive microcirculation protocol. Its potential value lies in making uncertainty and revision observable. The record shows which layer failed to recover, which mechanism was suspected, what response was predicted, when reassessment was due, and why escalation stopped or changed. These features can be measured even in centers that do not use direct microvascular imaging.

The framework cannot substitute for expertise or convert weak evidence into strong recommendations. Standardized CPB, avoidance of sustained inadequate oxygen delivery, patient blood management, temperature and acid-base control, echocardiographic diagnosis, surgical correction, and multidisciplinary coordination remain the foundation (18, 33, 35). A Tier 0 classification should be described as a suspected mismatch pattern, not direct proof of microvascular dysfunction.

Important limitations remain. The constructs were not derived from original data and may prove redundant, unstable, or clinically unhelpful. HVM, NIRS, venous Doppler, systemic hemodynamics, and metabolism sample different tissues and time scales; sublingual findings may not represent kidney, brain, or myocardium. A response to treatment is mechanistically non-specific and vulnerable to regression to the mean, concurrent interventions, and confirmation bias. Phenotypes may overlap, and apparent utilization failure is particularly difficult to establish.

Implementation also risks cognitive and treatment burden. The requirement to state a mechanism may encourage premature closure unless an indeterminate option and disconfirming evidence are explicit. Treatment-as-test can cause harm if it becomes an excuse for unnecessary fluid, vasopressor, transfusion, or delay in surgical correction. The workflow therefore applies only after urgent threats are addressed, only to interventions already judged clinically reasonable, and only with predefined stopping rules. Whether these safeguards work is an empirical question.

The targeted evidence map was not systematic and may have missed relevant studies. No pooled estimate or formal certainty rating was produced. The tiered workflow and validation sequence make the theory more testable, but they do not supply the missing validation. Until prospective reliability, safety, and comparative utility are demonstrated, 4D-Micro should remain a research framework and a transparent aid to clinical reasoning, not a mandated treatment algorithm. Publication of this theoretical framework should therefore be understood as publication of a specification for testing, not as evidence of predictive validity, treatment benefit, or readiness for clinical implementation.

## 10 Conclusion

4D-Micro is an unvalidated proposal for organizing discordance among systemic, regional, microvascular, and metabolic recovery during cardiac surgery. It does not replace conventional safety targets or require every center to measure all four domains. Its intended contribution is a tiered record of state, provisional mechanism, uncertainty, predicted response, and stopping rule.

The next step is not routine adoption but prospective falsification. Feasibility and interobserver reliability should be established before predictive validity or clinical utility is tested. Failure at any stage should lead to revision, narrowing, or rejection of the framework.

## Data Availability

No original patient or animal datasets were generated or analyzed for this article. The conceptual framework and supporting materials are included in the article and its Supplementary Material. Further inquiries can be directed to the corresponding author.

## Supplementary material

Supplementary Tables S1–S6 Targeted evidence-mapping strategy, construction rules, minimum episode record, candidate data dictionary, protocol-ready hypotheses, and a worked hypothetical episode for the 4D-Micro framework. No original patient or animal data are included.
