# Supplementary material for: 4D-Micro: a tiered, testable framework for hemodynamic-tissue perfusion mismatch in cardiac surgery
Source: Front Cardiovasc Med. 2026 Sep 30;13:1964275. doi: 10.3389/fcvm.2026.1964275 (PMC13661428; doi:10.3389/fcvm.2026.1964275)
Supplement: Supplementary file 1 [file Datasheet1.docx]

**Supplementary Material**

*Manuscript ID 1964275 | 4D-Micro: A Tiered, Testable Framework for Hemodynamic-Tissue Perfusion Mismatch in Cardiac Surgery*

**Authors: Budanbaila La1, Yanyan Bai2, Yiri Du1, Yi Qiu3, Ling Wang1***
This supplement contains no new patient or animal data.

# Purpose and status

This supplement makes the theory-construction process and proposed validation pathway auditable. It does not retrospectively convert the targeted evidence map into a systematic review, provide empirical validation, or authorize clinical treatment. The worked episode is hypothetical and is included only to demonstrate the proposed documentation logic.

**Supplementary Table S1. Targeted evidence-mapping strategy**

| **Element** | **Specification** |
| --- | --- |
| Databases | PubMed/MEDLINE and Cochrane Library; reference-list checking of guidelines, systematic reviews, pivotal trials, and recent reviews |
| Last update | 5 August 2026 |
| Population/context block | cardiac surgery OR cardiopulmonary bypass OR CPB OR off-pump coronary artery bypass OR OPCAB |
| Physiology block | microcirculation OR microvascular perfusion OR hemodynamic coherence OR tissue oxygenation OR oxygen extraction OR mitochondrial dysfunction |
| Barrier/conduit block | glycocalyx OR endothelial barrier OR permeability OR edema OR capillary leak |
| Driving/congestion block | cardiac output OR pump flow OR mean arterial pressure OR venous congestion OR central venous pressure OR venous Doppler |
| Rheology block | hemodilution OR hemoglobin OR oxygen delivery OR red-cell deformability OR microthrombosis OR transfusion OR patient blood management |
| Observation/intervention block | NIRS OR handheld vital microscopy OR HVM OR P(v-a)CO2 OR lactate OR vasoplegia OR ultrafiltration OR goal-directed perfusion |
| Priority | Adult cardiac-surgery evidence; current guidelines; systematic reviews; randomized trials; event-linked physiologic studies; critical-care evidence only when it clarified a transferable principle |
| Purpose and limitation | Theory construction and boundary checking, not exhaustive screening, pooled treatment estimation, or certainty grading |

**Supplementary Table S2. Construction rules and rejected overclaims**

| **Rule** | **Operational consequence** | **Rejected overclaim** |
| --- | --- | --- |
| Functional non-redundancy | Each domain represents a necessary exchange function that cannot be replaced by another | Four statistically proven latent variables |
| Location-cause separation | Coherence state identifies where recovery diverges; phenotype proposes why | A state is a diagnosis or unique mechanism |
| Directional response | A retained phenotype must predict a proximal response to a safe, clinically indicated, bounded test | Response proves causality |
| Explicit uncertainty | Mixed and indeterminate classifications are allowed; missing variables lower confidence | Every episode can be classified |
| Evidence separation | Mechanistic plausibility, proximal response, and patient-important benefit are reported separately | A plausible mechanism justifies routine treatment |
| Safety primacy | Urgent surgical/mechanical causes and established standards precede framework use | Framework replaces conventional monitoring or expertise |

**Supplementary Table S3. Minimum episode record**

| **Field** | **Minimum entry** | **Why it matters** |
| --- | --- | --- |
| Context | Procedure; CPB/OPCAB phase; operative event; timestamp | Defines the perturbation and plausible recovery window |
| Baseline | Patient baseline or immediately pre-event value | Supports within-patient rather than universal-threshold interpretation |
| Systemic layer | MAP plus pump flow or CO/CI when available | Documents upstream pressure and forward flow |
| Oxygen carriage | Hemoglobin/hematocrit, arterial oxygenation, temperature, acid-base status | Defines major delivery determinants and confounders |
| Downstream/non-systemic layer | At least one regional, congestion, metabolic, or organ observation when available | Required for cross-layer comparison; absence lowers confidence |
| Urgent exclusions | Artifact, bleeding, circuit failure, mechanical obstruction, RV compression, tamponade, arrhythmia, ischemia, ventilation | Prevents framework use from delaying definitive correction |
| Working label | State, phenotype, confidence, mixed/indeterminate status | Makes uncertainty explicit |
| Bounded test | Intervention already judged clinically indicated; dose/duration | Prevents diagnostic treatment solely for classification |
| Prediction | Expected proximal response, reassessment window, stop/toxicity boundary | Creates a falsifiable episode-level claim |
| Observed response | Direction, magnitude where measured, concurrent interventions, next decision | Updates rather than confirms the working explanation |

**Supplementary Table S4. Candidate data dictionary and timing**

| **Variable group** | **Candidate variables** | **Timing principle** | **Status** |
| --- | --- | --- | --- |
| Systemic | MAP, heart rate, rhythm, CO/CI, pump flow, SVR where available | Continuous or event-marked; summarize baseline, nadir, duration, recovery | Core |
| Oxygen transport | Hb/Hct, SaO₂, PaO₂, DO₂i, temperature, acid-base status, SvO₂/ScvO₂ | At baseline and clinically obtained samples around key events | Core/available |
| Regional | Cerebral/somatic NIRS, ST change, wall motion, organ-specific signals | Immediately before, during, and after defined event when feasible | Enhanced |
| Congestion | CVP, echo findings, venous Doppler, fluid balance, lung-water estimate | Event-linked and serial postoperative reassessment | Core/enhanced |
| Microvascular | HVM density, flow, velocity, heterogeneity; quality score | Synchronized research windows; blinded quality-controlled analysis | Research |
| Metabolic/organ | Lactate, P(v-a)CO₂, oxygen extraction, urine output, creatinine, neurologic or respiratory recovery | Use marker-appropriate delay; do not impose one universal window | Core/enhanced |
| Treatment | Fluid, blood, vasoactive/inotropic dose, pump adjustment, ventilation, temperature, mechanical/surgical action | Exact timestamp and concurrent changes | Core |

**Supplementary Table S5. Protocol-ready hypotheses and falsifiers**

| **Claim** | **Prediction** | **Comparator** | **Falsifier** |
| --- | --- | --- | --- |
| States can be recognized | Independent clinicians classify most eligible episodes with acceptable agreement and manageable time | Independent blinded classification | Low agreement, excessive time, or predominance of indeterminate labels |
| State and phenotype are distinct | The same coherence state can have different response patterns by phenotype, and one phenotype can occur across states | Alternative one-axis classification | No reproducible separation or redundant labels |
| Trajectory adds information | Duration, recurrence, and recovery slope add calibrated information beyond nadir/peak values | Baseline risk plus conventional time-varying variables | No incremental information or unstable effects across centers |
| Predicted response updates mechanism | Mechanism-concordant proximal responses occur more often than responses predicted by competing labels | Competing prespecified phenotype predictions | Similar response frequency across labels or strong dependence on unmeasured concurrent treatment |
| Workflow improves decisions | Less time in unresolved discordance and fewer repeated ineffective/contradictory interventions | Protocolized usual care with the same safety monitoring | No improvement or greater delay/intervention burden |
| Workflow is safe | No increase in ischemia, arrhythmia, hypotension burden, fluid overload, transfusion, or delayed mechanical correction | Protocolized usual care | Any reproducible increase in harm or delay |

**Supplementary Table S6. Worked hypothetical episode (not patient data)**

| **Step** | **Illustrative entry** | **Interpretation** |
| --- | --- | --- |
| Event | Five minutes after cardiac displacement during OPCAB, MAP and CI fall; cerebral NIRS decreases from the immediately pre-event baseline | Event-linked systemic and regional deterioration |
| Verify/exclude | Arterial waveform confirmed; ST segment and TEE reviewed; no major bleeding or arrhythmia; RV compression and reduced venous return are present | Mechanical cause has priority |
| Working classification | Indeterminate during acute systemic and regional deterioration; suspected low-driving/mechanical mechanism | Provisional location and cause are recorded separately |
| Bounded test | Reduce traction and reposition the heart; predicted response is recovery of CI and NIRS toward pre-event values within minutes; stop boundary is persistent ischemia or failure to recover | No drug or fluid is given solely to classify the episode |
| Observed response | CI and NIRS recover after repositioning | Response supports but does not prove the mechanical low-driving explanation |
| If response absent | Reassess graft-related ischemia, RV function, volume status, and conversion threshold; reclassify as mixed or indeterminate | The workflow prevents repeated nonspecific escalation |

**Caution:** This episode is illustrative, contains no real patient data, and does not define a treatment recommendation or universal reassessment interval.

# Link to main-manuscript evidence

Reference numbers and evidence summaries are provided in the main manuscript. The principal methodological addition is the staged development logic described by Skivington et al. (BMJ 2021;374:n2061; doi: 10.1136/bmj.n2061).
